# Prompt mental health care, the Norwegian version of IAPT: clinical outcomes and predictors of change in a multicenter cohort study

**DOI:** 10.1186/s12888-018-1838-0

**Published:** 2018-08-16

**Authors:** Marit Knapstad, Tine Nordgreen, Otto R. F. Smith

**Affiliations:** 1 Department of Health Promotion, Norwegian Institute of Public Health, Zander Kaaes gate 7, 5018 Bergen, Norway; 2 Department of Clinical Psychology, University of Bergen, Bergen, Norway; 3 Division of Psychiatry, Haukeland University Hospital, Bergen, Norway

**Keywords:** Prompt mental health care, IAPT, Anxiety, Depression, CBT

## Abstract

**Background:**

Prompt mental health care (PMHC) is a Norwegian initiative, inspired by the English ‘Improving Access to Psychological Therapy’ (IAPT), aimed to provide low-threshold access to primary care treatment for persons with symptoms of anxiety and depression. The objectives of the present study are to describe the PMHC service, to examine changes in symptoms of anxiety and depression following treatment and to identify predictors of change, using data from the 12 first pilot sites.

**Methods:**

A prospective cohort design was used. All participants were asked to complete questionnaires at baseline, before each treatment session and at the end of treatment. Effect sizes (ES) for pre-post changes and recovery rates were calculated based on the Patient Health Questionnaire and the Generalized Anxiety Disorder scale. Multiple imputation (MI) was used in order to handle missing data. We examined predictors through latent difference score models and reported the contribution of each predictor level in terms of ES.

**Results:**

In total, *N* = 2512 clients received treatment at PMHC between October 2014 and December 2016, whereof 61% consented to participate. The changes from pre- to post-treatment were large for symptoms of both depression (ES = 1.1) and anxiety (ES = 1.0), with an MI-based reliable recovery rate of 58%. The reliable recovery rate comparable to IAPT based on last-observation-carried-forward was 48%. The strongest predictors for less improvement were having immigrant background (ES change depression − 0.27, ES change anxiety − 0.26), being out of work at baseline (ES change depression − 0.18, ES change anxiety − 0.35), taking antidepressants (ES change anxiety − 0.36) and reporting bullying as cause of problems (ES change depression − 0.29). Taking sleep medication did on the other hand predict more improvement (ES change depression 0.23, ES change anxiety 0.45).

**Conclusions:**

Results in terms of clinical outcomes were promising, compared to both the IAPT pilots and other benchmark samples. Though all groups of clients showed substantial improvements, having immigrant background, being out of work, taking antidepressant medication and reporting bullying as cause stood out as predictors of poorer treatment response. Altogether, PMHC was successfully implemented in Norway. Areas for improvement of the service are discussed.

## Background

In Norway, anxiety and depression occupy the 8th and 9th positions among the most common causes of burden of disease [1]. However, access to mental health care services for persons with anxiety and depression is limited, and the treatment gap in Norway [2], as in many other European countries [3–5], is estimated to be over 50% for these disorders. In addition, though clients have a three-fold preference for psychological versus pharmacological treatment [6, 7], the latter is most often prescribed. The Organization for Economic Co-operation and Development (OECD) has urged Norway to address these weaknesses in care provision, in particular concerning the treatment of clients with mild to moderate anxiety and depression [3]. Currently, this group of clients is to a large degree dependent on care available from general practitioners (GPs).

In order to address the treatment gap, the UK government in 2007 announced the innovative, large-scale initiative “Improving Access to Psychological Therapy” (IAPT). In short, the initiative included an expansive training of new therapists to offer evidence-based psychological therapies as recommended by the National Institute of Clinical Evidence (NICE), organized consistent with a stepped care model. The scale-up was argued to be cost-effective by reducing welfare costs and increasing productivity [8, 9]. Following promising results from pilot studies [10, 11], the IAPT program was broadly rolled out in England in 2010. The program is continuously monitored, and the latest annual report found an average recovery rate of 49.3% and reliable recovery rate of 47.0% [12].

As each health care system is different, it is vital to illuminate whether large-scale implementation of the program is viable also in other countries. Thus far, the success of the IAPT initiative has encouraged replications in New Zealand and Australia. The Australian adaptation is evaluated finding promising results [13].

Prompt Mental Health Care (PMHC; “Rask Psykisk Helsehjelp” in Norwegian) service, is the Norwegian adaptation of IAPT. The Norwegian Ministry of Health and Care Services initiated PMHC as a pilot project at 12 sites in 2012, with the aim to increase access to evidence-based primary care treatment for adults with mild to moderate anxiety and depression [14]. As previously described [15], PMHC and IAPT share key characteristics by offering a free of charge, low-threshold service, aiming for short waiting times, and allowing for access without referral from the GP. In PMHC cognitive behavioural therapy (CBT) is provided, both as low- (guided self-help and group-based psychoeducation) and high-intensity (face-to-face) treatment. PMHC is organized according to a so-called matched-care model, in which information from the initial assessment and client preferences is used to determine the choice of treatment. This indicates, different from the stepped care model used in IAPT, that the client does not necessarily start with low-intensity treatment [16]. So far, PMHC has been established at 49 sites in municipalities and boroughs throughout Norway.

PMHC is evaluated using the same main outcome measures as in IAPT, more specifically the Patient Health Questionnaire Depression Scale (PHQ-9, [17]) and the Generalized Anxiety Disorder scale (GAD-7, [18]), to allow for cross-country comparisons. Initial results, presented in a research letter in “Psychotherapy and Psychosomatics” [15], show a recovery rate of 57%, reliable recovery rate of 52% and promising effect sizes (0.8–1.2) for pre-post improvement in symptoms of both anxiety and depression among the 12 first pilot sites.

The current paper will provide a more elaborate description of the PMHC service and show updated clinical outcomes based on a larger sample of PMHC users. In addition to provide updated effect sizes and recovery rates, the current paper will report predictors of clinical improvement from pre- to posttreatment. Though the effect of CBT is widely demonstrated [19–22], we need to know whether a large-scale implementation of this novel treatment model, focusing on low-intensity care delivered by multidisciplinary teams, is appropriate for clients with different socio-demographic backgrounds and clinical characteristics. In general, a vast number of studies have investigated factors predicting treatment response of CBT for anxiety and depression, however notably few consistent predictors are identified [23–25]. Pre-treatment severity is one of the most frequently reported predictor of poorer treatment outcome [19, 26], though its impact on degree of improvement is less clear [25, 27]. Among IAPT clients, disability, unemployment, younger age and functional impairment were associated with persistence of depression symptoms, and co-morbid depression and low outcome expectancy with persistence of anxiety symptoms after treatment [28]. Personality difficulties are found associated with less improvement in IAPT [29].

The lack of consistency with regard to predictors of treatment response mentioned above may partly be explained by the variation in analytic strategies. It is known that using categorical outcomes versus continuous outcomes and/or simple gain scores versus residualized changes scores can influence findings. More importantly, the bulk of evidence emerge from comparably small trials in controlled settings, which often have strict inclusion criteria and insufficient power to examine treatment effects across groups [25]. Predictor studies from routine care, large-scale implementations may therefore be of interest as these both ensure sufficient statistical power and increase the generalizability of findings. On the downside, the latter type of studies are typically based on single-group observational study designs, which by nature complicates causal inference. Use of simple gain scores provide unbiased estimates of differential treatment effects if the assumption of a stable base rate in the control group holds [30, 31]. In the absence of a control group, it is impossible to test this assumption. However, there is evidence from previous studies that clients in waitlist control conditions with a prior duration of clinically significant anxiety and/or depression for six months or over tend to report low to very low recovery rates (5–20%) [32–34]. Therefore, in PMHC clients with a prior duration of 6 months or more, it may be reasonable to assume that depression and anxiety scores would continue to be relatively stable without any form of treatment [10]. As such, analyses based on simple gain scores in this subgroup of clients may point to differential treatment effects of relevant demographic factors and clinical characteristics.

In summary, the present paper extends findings from a report in Norwegian [35] and a research letter in ‘Psychotherapy and Psychosomatics’ [15], and has the following objectives: 1) To describe the general characteristics of PMHC. 2) To elaborate on the main findings with regard to effect sizes of clinical improvement and recovery rates from pre- to post- treatment. 3) To examine the predictive value of a range of baseline characteristics for treatment response. Merged data from the first 12 pilot sites will be employed for all analyses.

## Methods

### Pilot sample

The first 12 PMHC pilot sites were established in 2012–2013. The sites were distributed across several geographical areas in eastern, western and central Norway, including both urban and rural areas. Nine of the pilot sites were located in individual municipalities (Fjell, Hurum, Kristiansund, Lørenskog, Modum, Molde, Notodden, Orkdal and Stjørdal), one through inter-municipal cooperation (Fosen DM IKS) and two covered boroughs in the Oslo municipality (Frogner and Søndre Nordstrand). The population size varied from 11,722 in rural Orkdal to 55,965 in urban Oslo Frogner. The demographic profiles of the pilot sites displayed notable differences as well. For example, the proportion of inhabitants with an immigration background varied from 5.8% in Fosen DM IKS to 52.2% in Oslo Søndre Nordstrand and the proportion of persons on permanent disability pension varied from 4.5% in Oslo Frogner to 14.4% in Notodden [35].

The PMHC teams had on average four full-time equivalents independent of the catchment area population size. All teams were multidisciplinary and had at least one clinical psychologist who carried the professional responsibility for the services provided. All therapists had a minimum of three years with relevant higher education and completed an additional, mandatory one-year training in cognitive behavioural therapy under the auspices of the Norwegian Association for Cognitive Therapy. The curriculum was based on IAPT, but adjusted to the Norwegian context. It included 208 h of tutoring and 96 h of supervision from clinical psychologists, in addition to peer supervision. All therapists had individual treatment responsibilities.

### Procedures

All clients contacting PMHC participated in an initial assessment. During this session, information about the content and treatment methodology within PMHC was provided, and the therapist assessed relevant information to decide whether PMHC could be the appropriate treatment or not. The therapist identified the relevance and severity of the mental problems, and the available client resources.

#### Treatment inclusion criteria

Inhabitant of the pilot site community, ≥18 years of age, anxiety disorder and/or mild to moderate symptoms of depression (formal diagnosis not provided).

#### Treatment exclusion criteria

Clients with history or clear indications of psychosis, bipolar disorder, personality disorder, severe drug abuse, and suicide risk were generally excluded from PMHC, and were referred to the GP or secondary health care services.

Participation was based on opt-in, where clients who were suitable for treatment were informed about the study, invited to participate and asked to sign an informed consent. The participants were asked to complete questionnaires before the first treatment session, before each session during the treatment, and at post-treatment. In more than 97% of the cases, participants completed the questionnaires electronically. For each participant, the therapists (*n* = 68) were asked to complete a questionnaire at post-treatment about the therapy process.

The study was approved by the Regional Ethics Committee for Western Norway (REK-vest no. 2014/597).

### Participants

**Fig. 1:**
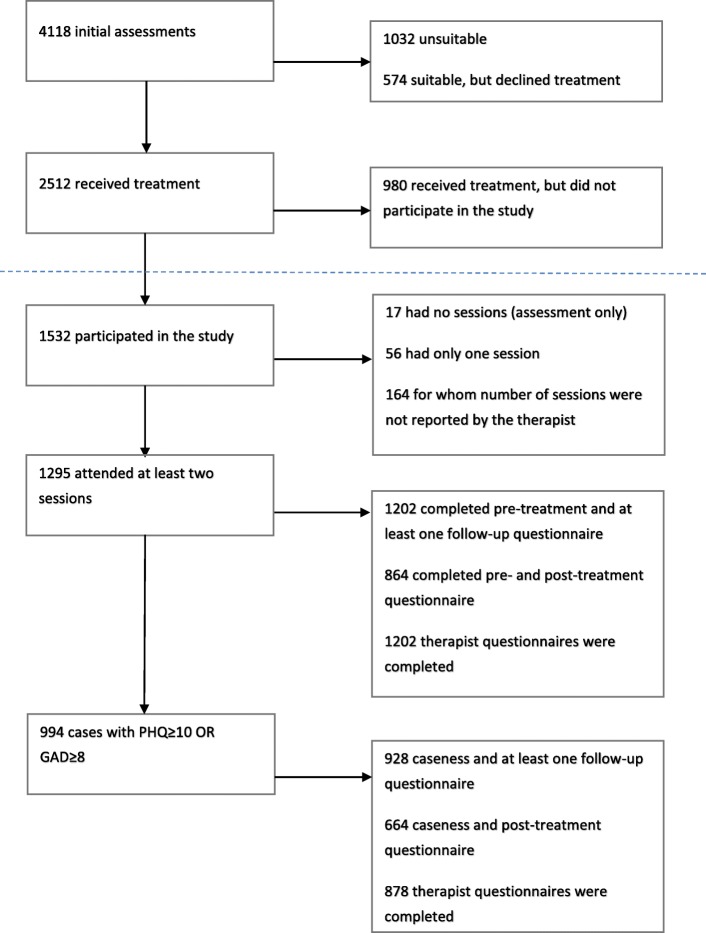
Flow diagram of PMHC for period October 2014 to December 2016

As displayed in Fig. 1, 2512 clients started treatment at PMHC between October 2014 and December 2016. This number varied between 88 in Stjørdal and 395 in Lørenskog [35]. Of those who started treatment, 1532 (61%) signed informed consent. The study participation rate varied between 27.7% in Orkdal to 79.3% in Oslo Frogner. In 8 out of 12 pilot sites, the participation rate was over 60%. Of the 1532 participating clients, 1297 had attended at least two sessions. Number of sessions does not include initial assessment since the clients were included in the study after this session.

### Missing data

Missing data rates at baseline were generally low (< 5%) with exception of weight (10.2%), antidepressant medication (11.9%), anxiolytic medication (10.4%), sleep medication (10.4%), and life events (10.2%). For the overall sample, 21.3% of the cases did not have any follow-up assessment, while post-treatment scores for PHQ and GAD were missing for 43.6% of the cases. For the sample who attended at least two sessions, 7.2% of the participants did not have a follow-up assessment, while post-treatment scores for PHQ and GAD were missing for 33.2% of the participants. In both groups, missing data for PHQ and GAD at post-treatment were associated with being younger, higher mean scores for the last observed score on PHQ and GAD while under treatment, and the following self-reported causes of current mental health problems: difficult childhood and having been bullied (all *p* < .05). Missingness in the overall sample was also associated with low educational level, being a smoker, and the following self-reported causes of current mental health problems: romantic relationship problems and family relationship problems (all *p* < .05). This may suggest that missing data at post-treatment were (partly) “missing at random” (MAR) [36]. It should also be noted that 25.0% of the variance in missing scores at final treatment was explained at the therapist level, while this was the case for less than 3% of the variance in PHQ/GAD changes scores. With the exception of the “under treatment” PHQ/GAD data, the other missing data correlates were only weakly associated with PHQ/GAD at post-treatment. This implies that omitting these variables from the (missing) data analysis model would not bias PHQ/GAD estimates at post-treatment. It is nonetheless likely that in these types of settings part of the missing data is “missing not at random” (MNAR) as well [36]. Nonresponse for some participants may be more likely because of the actual (but unobserved) PHQ/GAD scores at post-treatment, which is conceivable for both those who are not improving and those recovering. The bias introduced by MNAR can partly be eliminated by including strong correlates of variables with missing data. In the present study, there was a relatively strong relationship between the observed PHQ and GAD scores at post-treatment and respectively baseline PHQ and GAD scores (r ≈ .4) and the last observed scores on PHQ and GAD while under treatment (r ≈ .6). Finally, some of the missing data may also be “missing completely at random” (MCAR) [36]. Information from the therapists indicated that missing questionnaires were often due to lack of time or the result of participants forgetting to complete the questionnaire.

### Measures

The Patient Health Questionnaire (PHQ-9) was used to measure depressive symptoms [17]. It includes nine items based on each of the DSM-IV criteria for depression, with response categories range from 0 (“not at all”) to 3 (“nearly every day”). This yielded a total sum score that ranged from 0 to 27. The PHQ has good psychometric properties [17]. Cronbach’s alpha based on PMHC data was 0.85.

The Generalized Anxiety Disorder Assessment (GAD-7) was used to measure symptoms of anxiety disorder [18]. It includes seven items to score common anxiety symptoms ranging from 0 (“not at all”) to 3 (“nearly every day”). Total score could range from 0 to 21. In addition to measuring generalized anxiety disorder, there are indications that the GAD-7 also has good sensitivity and specificity for panic, social anxiety, and post-traumatic stress disorder [18]. Cronbach’s alpha based on PMHC data was 0.87.

Participants were defined as caseness when scoring PHQ ≥ 10 and/or GAD ≥8.

The following self-reported baseline characteristics were included as potential predictors of change:

#### Socio-demographic factors

Gender, age, educational level (primary school, secondary school, higher education), marital status (having a partner, not having a partner), immigration background (defined as being an immigrant or born in Norway with immigrant parents). Employment status was assessed by means of two questions, one multi-response item about employment status, and one multi-response item about sources of income [35]. Based on these two questions, participants were placed into three categories: 1) In regular work, 2) In combined work and a recipient of benefits (graded sick leave, disability, unemployment or other benefits), and 3) Out of work with or without benefits. A similar categorization has been used in another Norwegian treatment study [37].

#### Life style and social factors

Physical activity (days per week), BMI, smoking (yes, no), alcohol use (2–3 times a week or more, less than 2–3 times per week).

Social support was assessed using the Oslo 3-items social support scale (OSS-3) [38]. The items cover number of close confidants, the sense of concern or interest shown by others and perceived availability of practical help from neighbors. Due to the relatively low internal consistency and the subjective nature of the items in the PMHC data (Cronbach’s alpha = .64), social support was modelled as a latent variable.

Life-events were measured by means of the Life-events scale [39], which screens for 24 specific life-events that the participant is asked to rate the impact of from − 3 (very negative) to 3 (very positive). A sum-score for the total impact of life-events was made by merging the impact of negative and positive events.

#### Other relevant factors

Duration of psychological problems prior to the initial assessment (< 6 months, ≥6 months), referral (self, health personnel), previous treatment attempts by psychologist or psychiatrist last 12 months (yes, no), use of antidepressant medication (every day, less than every day), use of anxiolytic medication (every week, less than every week), use of sleep medication (every week, less than every week), perceived cause of symptoms (relationship problems, family relationship problems, school/job related, difficult childhood and/or bullying: yes, no).

Number of sessions and length of treatment (log-transformed due to positive skewness) were included in sensitivity analyses as predictors of change in the multivariate predictor model, to examine their impact on the predictive values of the baseline variables.

### Statistical analyses

Basic descriptive analyses were carried out using Stata Version 15.0. All other analyses were conducted in Mplus Version 8. For all analyses in Mplus, type = complex was used to account for clustering within pilot sites.

Multiple imputation (MI) was used to handle missing data in predictor and outcome variables. All predictor variables mentioned above, the variables containing the last available PHQ and GAD scores during treatment, and the post-treatment variables for PHQ and GAD were included in the imputation model. The multiple imputation procedure in Mplus was used to generate 20 imputed datasets. MI is valid under the MAR assumption and is generally preferred over more traditional methods for dealing with missing data.

For observed sum scores, empty means models with an unstructured R-matrix were used to estimate means at pre- and post-treatment in symptoms of depression (PHQ-9) and anxiety (GAD-7). Unadjusted effect sizes were calculated by dividing the mean difference score by the standard deviation at pre-treatment (Cohen’s *d*) using the model constraints command in Mplus.

In addition to observed sum scores, we also reported the results from multiple indicators latent difference score models [40]. The advantage over the more traditional approach based on observed sum scores is that latent difference scores reflect meaningful differences rather than differences that (partly) result from measurement error, and reduces regression to the mean effects. As a first step, measurement invariance across time (i.e. pre-post) was tested for PHQ-9 and GAD-7 (MLR estimator, continuous indicators). The configural model yielded an acceptable fit for both PHQ-9 (RMSEA = .06, CFI = .94) and GAD-7 (RMSEA = .06, CFI = .97). Changes in model fit were minimal when testing the metric vs configural (PHQ: ΔRMSEA = .001, ΔCFI = −.006; GAD: ΔRMSEA = .002, ΔCFI = −.006) and scalar vs metric models (PHQ: ΔRMSEA = 0, ΔCFI = −.005; GAD: ΔRMSEA = 0, ΔCFI = −.003).

The primary analyses for changes from pre- to post-treatment were carried out for participants who attended at least two sessions (*n* = 1295).

Sensitivity analyses were conducted by means of the conservative last observation carried forward (LOCF) method for those who attended at least two sessions (*n* = 1295) and the liberal listwise deletion (LD) method for those who completed both the pre- and post-treatment questionnaire (*n* = 864). For LOCF the initial PHQ/GAD scores and the last available PHQ/GAD score before discharge were used. Additionally, Intention to treat analyses using both MI (*n* = 1532) and LOCF (*n* = 1519, due to missing at all time points for *n* = 13) were carried out. For the participants who completed at least two sessions, additional subgroup analyses were conducted. Effect sizes were calculated for those that started treatment at caseness (*n* = 994) and those that reported having mental problems at least six months prior to treatment (*n* = 1059).

Recovery rates were calculated based on observed sum scores. Recovery was defined as scoring above the caseness threshold on the PHQ-9 (≥ 10) and/or GAD-7 (≥ 8) measures at the start of treatment and below the caseness threshold on both these measures at the end of the treatment. Reliable recovery rate was calculated in order to account for measurement error, aligning with the procedures employed for the IAPT evaluations [41]. Using the standard deviation of the sample and Cronbach’s alpha for PHQ and GAD, a reliable change score of ≥6 was derived for PHQ and ≥ 5 for GAD.

The predictor analyses were performed in the sample that had completed at least two sessions and reported having problems at least six months prior to treatment (*n* = 1059). The latter restriction was chosen to limit the impact of natural recovery or regression to the mean effects, which may be less pronounced for longer-lasting problems [32–34]. Predictors were examined through multiple indicator latent difference score models and the contribution of each predictor level was expressed in terms of ES. The predictors were first examined separately. Those predicting change at an ES level of ≥0.1 were included in a multivariate model.

## Results

### Demographic and clinical characteristic of participants

Compared to the population statistics of the PMHC catchment areas [42], males (25.5%), older individuals (> 67 years = 1.2%), those with lower education (10.2%), and immigrant background (11.3%) were underrepresented among study participants (Table 1). This pattern was observed across all pilot sites. The subgroups who attended at least two sessions and those who attended two sessions and had pre-treatment symptoms ≥6 months were in large similar to the overall sample (Table 1), except those having at least two sessions were somewhat higher educated and fewer were out of work (*p* < 0.001). The proportion of participants with problem duration of six months or longer prior to the initial assessment was 84.3%. In all pilot sites, this proportion was above 75.0%. Mean baseline scores for PHQ-9 were 12.5 (SD = 5.7) and for GAD-7 10.1 (SD = 5.0). Split by urban and rural sites, baseline GAD-7 score were slightly higher at the urban (10.5 (SD5.0)) than at the rural sites (mean 9.9 (SD 5.0) (*t* = − 2.2, df = 1514, *p* = 0.032). There were, however, no difference in neither baseline PHQ-9 score nor percentages of participants with PHQ-9 or GAD-7 scores that can be classified as severe (i.e. ≥29 for PHQ-9 and ≥ 15 for GAD). Using the pre-defined cut-offs for PHQ and GAD, 77.2% of the participants could be identified as being at caseness at pre-treatment. The variation across pilot sites with regard to the percentage of caseness at pre-treatment varied between 70.1% (Fosen DM IKS) and 83.2% (Notodden). In 45.2% of the cases, the therapists registered depression as the primary provisional diagnosis, 21.5% were registered for a specific anxiety disorder, and in 20.2% of the cases mixed depression and anxiety was indicated (see also Table 2).

**Table 1:** Demographic characteristics of PMHC clients who participated in the study, in total and for each sub-sample analysed^a^

Variable	Total sample (*n* = 1532)	≥ 2 sessions (*n* = 1295)	≥ 2 sessions and problems ≥6 months (*n* = 1059)
	% (n)	% (n)	% (n)
Female sex	74.5 (1127)	74.2 (951)	73.8 (775)
Age group			
18–25 years	16.1 (244)	15.2 (195)	14.0 (148)
26–44 years	54.0 (817)	54.4 (698)	54.7 (577)
45–67 years	28.7 (435)	29.1 (373)	30.0 (316)
> 67 years	1.2 (18)	1.3 (17)	1.2 (13)
Educational level			
Primary school	10.2 (154)	9.1 (116)	8.6 (90)
Secondary school	45.5 (688)	45.1 (578)*	46.0 (483)
Higher education	44.3 (669)	45.8 (587)***	45.5 (478)
Marital status			
Single	28.1 (424)	27.2 (348)	26.0 (273)
Married	32.4 (489)	32.7 (419)	33.4 (351)*
Living together	28.8 (435)	29.4 (377)	29.2 (307)
Divorced	9.6 (145)	9.7 (124)	10.6 (111)*
Widowed	1.1 (17)	1.0 (13)	0.9 (9)
Immigrant background	11.3 (170)	10.7 (138)	10.6 (112)
Employment status			
In regular work	38.9 (587)	39.8 (508)	39.7 (415)
Combined work and a recipient of benefits	35.4 (535)	36.0 (460)	34.8 (364)
Out of work with or without benefits	25.7 (389)	24.3 (310)**	25.5 (266)
Available follow-up data			
At least one follow-up questionnaire	78.7 (1205)	92.8 (1202)***	93.0 (985)***
Post-treatment questionnaire	56.4 (864)	66.7 (864)***	66.7 (706)***

^a^Dispersed numbers due to missing data. Range missing per variable: total sample, *n* = 18–24; those having ≥2 sessions, *n* = 3–17; those having ≥2 sessions and problem duration of ≥6 months, *n* = 1–14

Distribution different from the comparison sample at *p < 0.05, ** *p* < 0.01 and *** *p* < 0.001. Differences examined using logistic regression tests

**Table 2:** Clinical and treatment characteristics of PMHC clients who participated in the study, in total and for each sub-sample analysed^a^

	Total sample (*n* = 1532)	≥ 2 sessions (*n* = 1295)	≥ 2 sessions and problems ≥ 6 months (*n* = 1059)
Client-reported clinical and treatment characteristics			
Data completeness (%)	> 95%	> 95%	> 95%
Variable	Estimate	Estimate	Estimate
Duration of psychological problems prior to initial assessment ≥6 months, % (n)	84.3 (1228)	84.0 (1059)	100.0 (1059)
Previous treatment attempts last 12 months^b^	15.9 (244)	16.5 (213)	17.9 (190)*
Caseness at baseline, % (n)			
PHQ ≥ 10	67.2 (1019)	66.2 (856)*	67.2 (712)
GAD ≥8	65.0 (985)	64.5 (835)	64.6 (648)
PHQ ≥ 10 OR GAD ≥8	77.2 (1172)	76.8 (994)	77.4 (820)
Medication use, % (n)^c^			
Antidepressant, daily	13.6 (187)	13.9 (164)	14.2 (138)
Anxiolytic, weekly	8.9 (120)	8.4 (98)	8.5 (81)
Sleep, weekly	12.5 (171)	12.5 (148)	12.5 (120)
Therapist-reported clinical and treatment characteristics			
Data completeness, % (n)	75.1 (1150)	85.7 (1110)^d^	85.4 (905)
Variable	Estimate	Estimate	Estimate
Referral, % (n)			
GP or other health personnel	57.0 (655)	56.7 (629)	57.4 (519)
Self	43.0 (495)	43.3 (481)	42.7 (386)
Tentative primary diagnosis, % (n)			
Major depressive disorder	45.2 (520)	45.1 (500)	47.2 (427)**
Panic disorder with agoraphobia	4.1 (47)	4.1 (46)	4.2 (38)
Panic disorder without agoraphobia	4.6 (53)	4.7 (52)	3.5 (32)
Social anxiety disorder	5.2 (60)	5.1 (57)	5.4 (49)
Hypochondria	2.7 (31)	2.8 (31)	2.9 (26)
Generalized anxiety disorder	3.4 (39)	3.3 (37)	3.4 (31)
Posttraumatic stress syndrome (PTSD)	1.0 (12)	1.1 (12)	0.9 (8)
Obsessive compulsive disorder	0.5 (6)	0.5 (6)	0.3 (3)
Mixed anxiety and depression	20.2 (232)	20.4 (226)	20.2 (183)
Other	13.1 (150)	12.9 (143)	11.9 (108)
Waiting times, median (IQR)			
Days between initial contact and assessment	10.0 (4.0–22.0)	11.0 (4.0–23.0)	12.0 (4.0–24.0)
Days between assessment and first treatment session	8.0 (4.0–15.0)	8.0 (4.0–15.0)	8.0 (4.0–15.0)
Days between initial contact and first treatment session	22.0 (12.0–41.0)	22.0 (12.0–41.0)	23.0 (13.0–42.0)
Treatment duration, median (IQR)			
Number of attended sessions	6.0 (4.0–9.0)	6.0 (4.0–9.0)	6.0 (4.0–9.0)
Number of weeks	11.1 (5.4–19.0)	11.1 (5.4–19.0)	11.1 (5.8–19.3)
Type of treatment			
Proportion of total number of registered sessions by mode of treatment (≈7000 sessions), %			
Guided self-help	8.3	8.4	8.0
Group course psychoeducation	16.5	16.7	16.9
Face-to-face	71.3	72.1	72.1
Proportion of clients that during the course of treatment used mode of treatment, %			
Guided self-help	16.9	17.1	16.7
Group course psychoeducation	21.5	22.1	22.3
Face-to-face	76.6	77.8	77.3

^a^Dispersed numbers due to missing data

^b^by psychologist or psychiatrist

^c^Data completeness ≈ 90%.s

^d^Therapist questionnaires available for *n* = 1202 (92.8%), but information about the characteristics reported in this table was missing for *n* = 92

Distribution different from the comparison sample at *p < 0.05,** *p* < 0.01, and *** *p* < 0.001. Differences examined using chi-square tests

### Treatment characteristics of PMHC

As reported by the therapists, just above half of the participants were referred by GPs or other health personnel (57.0%), the rest were self-referrals (Table 2). Self-referral was less common among participants with immigrant background (32.3%, OR = 0.59 (95% CI 0.40–0.89)) and those in combined work and a recipient of benefits (38.7%, OR = 0.73 (95% CI 0.56–0.96)). No other significant associations were identified between type of referral and the pre-treatment characteristics (Table 1). This may suggest that self-referral did not contribute to increased participation of the underrepresented groups mentioned above. Those referred by health personnel had slightly higher mean PHQ-9 score at baseline (12.9 (SD = 5.67)) than the self-referrals (11.6 (SD = 5.3)) (*t* = 4.0, df = 1147, *p* < 0.001).

Median waiting time was 10.0 days between initial contact and assessment, and 22.0 days between initial contact and first treatment session. Waiting times varied considerably across pilot sites, with 8 days in Notodden and 48.0 days in Lørenskog between initial contact and first treatment session. The median treatment duration was 11.1 weeks and the median number of attended sessions was 6.0.

Based on the total number of registered sessions after the initial assessment, the majority of sessions were used on face-to-face treatment (71.3%). Moreover, 76.6% of the participants received at least one face-to-face session during the course of treatment. Guided self-help (8.3% of sessions) and group course psychoeducation (16.5% of sessions) were used less frequently (Table 2), although there were large variations across pilot sites [35]. Pilot sites that used guided self-help most frequently were Fosen DM IKS (30.8% of sessions) and Molde (35.7% of sessions), whereas group course psychoeducation was most common in Fjell (46.5%) and Notodden (31.2%).

### Clinical outcomes

Table 3 details the effect sizes of pre-post change, recovery rates and reliable recovery rates with 95% CIs for PHQ and GAD, including primary and sensitivity analyses as well as subgroup analyses for those with ≥2 sessions. Changes are in the expected direction with large effect sizes (ES) and recovery rates exceeding the 50% target used in IAPT for all estimates but the most conservative (ITT sample with LOCF missing data technique). In the latter, the ESs were still in the upper moderate range.

**Table 3:** Pre-post estimates for symptoms of depression (PHQ) and anxiety (GAD)

Analysis	Missing data technique^a^	N	ES (PHQ)	ES (GAD)	N clinical case	Recovery rate	Reliable recovery rate
Primary analyses							
Attended at least two sessions	MI	1295	1.09 (1.02,1.16)	1.03 (0.97,1.10)	994	65% (61%, 69%)	58% (54%, 61%)
Attended at least two sessions - Latent	MI	1295	1.20 (1.07, 1.32)	1.13 (1.02, 1.24)	–	–	–
Sensitivity analyses							
Intention to treat	MI	1532	1.09 (1.03, 1.15)	1.04 (1.00, 1.10)	1172	64% (60%, 67%)	57% (53%, 60%)
Intention to treat	LOCF	1519	0.74 (0.69, 0.79)	0.71 (0.64, 0.76)	1172	46% (43%, 49%)	41% (38%, 44%)
Attended at least two sessions	LOCF	1294	0.88 (0.82, 0.94)	0.83 (0.78, 0.88)	994	57% (54%, 61%)	48% (45%, 51%)
Completed pre- and post-treatment questionnaires	LD	864	1.13 (1.05, 1.21)	1.04 (0.97, 1.11)	663	69% (66%, 72%)	62% (59%, 66%)
Subgroup analyses for those with ≥2 sessions							
Started treatment at caseness	MI	994	1.53 (1.44, 1.63)	1.41 (1.32, 1.50)	994	65% (61%, 69%)	58% (54%, 61%)
Started treatment at caseness - Latent	MI	994	1.79 (1.59, 1.99)	1.62 (1.46, 1.77)	–	–	–
Pre-treatment symptoms > 6 months	MI	1059	1.08 (1.01, 1.15)	1.01 (0.94, 1.08)	820	64% (60%, 67%)	56% (52%, 60%)
Pre-treatment symptoms > 6 months - Latent	MI	1059	1.18 (1.04,1.32)	1.10 (0.98, 1.23)	–	–	–

^a^*MI* Multiple Imputation, *LOCF* Last Observation Carried Forward

More specifically, in the sample that attended at least two sessions and using MI to handle missing outcome data, the ES of the average observed change scores (pre minus post) were 1.09 (95% CI: 1.02, 1.16) for PHQ and 1.03 (95% CI: 0.97, 1.10) for GAD. Employing latent variable change score models, which exclude measurement error, the ESs were 1.20 for symptoms of depression and 1.13 for symptoms of anxiety.

Approximately the same estimates were found in both the ITT sample using MI and among those who completed both the pre- and post-treatment questionnaire, as the observed scores from the primary analysis. To set a lower bound of effect, sensitivity analyses using LOCF missing data technique gave observed change scores of ES = 0.88 and 0.74 for PHQ and 0.83 and 0.71 for GAD in the sample attended at least two sessions and the ITT sample, respectively.

Looking at those meeting criteria for caseness only, the ESs of improvement were markedly larger (1.53 for observed PHQ /1.79 latent PHQ score and 1.41 for observed GAD/1.62 latent GAD score). Restricting the analyses to participants with problem duration of > 6 months hardly changed the ES estimates.

The recovery rate was 65 (95% CI: 61%, 69%) and reliable recovery rate 58% (95% CI: 54%, 61%) in the sample that completed at least two sessions, using MI. Again, these estimates were quite similar to the results for the ITT sample using MI (64% and 57%) and for those who competed both the pre- and post-treatment questionnaires (69% and 62%). In the ITT sample when using LOCF (most conservative estimate), the recovery rate was 46% and reliable recovery rate 41%. The comparable IAPT estimates (LOCF and at least two sessions) were respectively 57% and 48%.

A sensitivity analysis showed that only 2.2% (*n* = 10) of the clients defined as pre-treatment caseness changed less than three points on the PHQ and/or GAD scales from pre- to post-treatment. The same was true for those having at least two sessions.

### Baseline predictors of change

Table 4 details the baseline characteristics that significantly predicted reliable change in latent depression and/or anxiety score from pre- to post-treatment (*p* < 0.05 and ES ≥ 0.1). The contribution of each predictor level is reported in terms of ES (95%CI), where negative numbers equals less improvement and positive more improvement on latent symptom scores. As mentioned in the statistical analyses section, clustering within pilot-sites is accounted for by using the Mplus function type = complex. For completeness, the impact of site on PHQ and GAD changes scores is determined by calculating intraclass correlations. These were 0.014 for PHQ and 0.012 for GAD, suggesting that little variation in change scores was explained by site.

**Table 4:** Predictors of change in latent PHQ and/or GAD score from pre- to post-treatment^*^

	PHQ			GAD		
	Baseline level^†^ SD (95% CI)	Reliable change Crude ES (95% CI)	Reliable change Adjusted ES (95% CI)	Baseline level^†^ SD (95% CI)	Reliable change Crude ES (95% CI)	Reliable change Adjusted ES (95% CI)
Gender						
Intercept	–	1.12 (0.98, 1.26)			1.02 (0.89, 1.15)	1.01 (0.73, 1.30)
Female gender	0.09 (0.02, 0.16)	0.08 (−0.05, 0.21)			0.11 (− 0.03, 0.26)	0.11 (− 0.03, 0.25)
Education						
Intercept	–	1.20 (1.02,1.39)	1.27 (1.05, 1.50)	–	1.11 (0.94, 1.29)	1.01 (0.73, 1.30)
Primary school	**0.42 (0.16, 0.68)**	−0.20 (− 0.50, 0.10)	− 0.15 (− 0.46, 0.16)	**0.23 (0.06, 0.40)**	−0.16 (− 0.41,0.08)	−0.00 (− 0.25, 0.25)
Secondary school	0.18 (− 0.01, 0.37)	−0.01 (− 0.21,0.19)	0.00 (− 0.20, 0.20)	0.14 (− 0.01, 0.29)	−0.00 (− 0.18, 0.18)	0.05 (− 0.12, 0.23)
Living alone						
Intercept	–	1.13 (0.96, 1.29)	1.27 (1.05, 1.50)	–	1.10 (0.95, 1.25)	
Yes	**0.18 (0.04, 0.33)**	**0.14 (0.00, 0.28)**	**0.16 (0.03, 0.28)**	0.09 (−0.16, 0.16)	0.00 (− 0.16, 0.16)	
Ethnicity						
Intercept	–	1.22 (1.09, 1.35)	1.27 (1.05, 1.50)	**–**	1.13 (1.02, 1.25)	1.01 (0.73, 1.30)
Immigrant background	**0.19 (0.02, 0.35)**	**−0.34 (− 0.66, − 0.02)**	**−0.27 (− 0.55, − 0.01)**	**0.36 (0.04, 0.44)**	− 0.29 (− 0.59, 0.01)	**−0.26 (− 0.51, − 0.01)**
Job status						
Intercept	–	1.20 (1.07, 1.33)	1.27 (1.05, 1.50)	–	1.18 (1.04,1.33)	1.01 (0.73, 1.30)
Job and support	**0.36 (0.24, 0.48)**	**0.15 (0.00, 0.29)**	0.13 (−0.00, 0.26)	0.10 (−0.02, 0.21)	0.03 (− 0.12, 0.18)	0.00 (− 0.13, 0.14)
No job with/without sup	**0.19 (0.03, 0.34)**	**−0.26 (− 0.41, − 0.12)**	**−0.18 (− 0.29, − 0.07)**	−0.07 (− 0.24, 0.11)	**−0.35 (− 0.51, 0.20)**	**−0.35 (− 0.50, − 0.21)**
Physical activity						
Intercept	–	1.35 (1.16, 1.55)	1.27 (1.05, 1.50)	–	1.15 (1.00, 1.30)	
Physical activity	**−0.16 (− 0.23, − 0.10)**	**−0.11 (− 0.18, − 0.03)**	**−0.11 (− 0.19, − 0.03)**	−0.03 (− 0.09, 0.04)	−0.02 (− 0.08, 0.04)	
Alcohol consumption						
Intercept	–	1.18 (1.03, 1.32)		–	1.08 (0.95, 1.21)	1.01 (0.73, 1.30)
High consumption	0.03 (−0.29, 0.23)	0.02 (−0.27, 0.32)		0.00 (−0.23, 0.23)	0.12 (− 0.14, 0.38)	0.13 (− 0.12, 0.38)
Previous treatment						
Intercept	–	1.22 (1.01, 1.36)	1.27 (1.05, 1.50)	–	1.13 (1.01, 1.26)	1.01 (0.73, 1.30)
Yes	0.04 (−0.17, 0.25)	**−0.21 (− 0.42, 0.00)**	−0.15 (− 0.33, 0.03)	−0.02 (− 0.22, 0.18)	**−0.18 (− 0.34, − 0.00)**	−0.11 (− 0.27, 0.05)
Antidepressant medic.						
Intercept	–	1.21 (1.07, 1.35)	1.27 (1.05, 1.50)	–	1.14 (1.01, 1.28)	1.01 (0.73, 1.30)
Every day	**0.36 (0.18, 0.55)**	−0.19 (−0.42, 0.04)	− 0.22 (− 0.41, − 0.02)	0.11 (− 0.11, 0.33)	**−0.27 (− 0.49, − 0.06)**	**−0.36 (− 0.56, − 0.16)**
Sleep medication						
Intercept	–	1.16 (1.03, 1.29)	1.27 (1.05, 1.50)	–	1.06 (0.93, 1.19)	1.01 (0.73, 1.30)
Every week	**0.46 (0.18, 0.74)**	0.13 (−0.08, 0.33)	**0.23 (0.02, 0.45)**	**0.48 (0.20, 0.77)**	**0.27 (0.11, 0.44)**	**0.45 (0.26, 0.64)**
Perceived cause						
Intercept	–	1.14 (0.99, 1.28)	1.27 (1.05, 1.50)	–	1.09 (0.97, 1.22)	
Relationship problems	**0.32 (0.22, 0.43)**	**0.12 (−0.00, 0.24)**	**0.15 (0.02, 0.29)**	**0.22 (0.05, 0.45)**	0.02 (− 0.15, 0.19)	
Perceived cause						
Intercept	–	1.09 (0.93,.1.24)	1.27 (1.05, 1.50)	–	1.06 (.93, 1.19)	
School/job related	**0.31 (0.17, 0.45)**	**0.20 (0.06, 0.33)**	**0.21 (0.10, 0.31)**	**0.18 (0.10, 0.27)**	0.09 (−0.05, 0.22)	
Perceived cause						
Intercept	–	1.22 (1.09, 1.36)	1.27 (1.05, 1.50)	–	1.13 (1.01, 1.25)	1.01 (0.73, 1.30)
Bullying	**0.27 (0.06, 0.48)**	**−0.33 (−0.62, − 0.04)**	**−0.29 (− 0.57, − 0.01)**	**0.28 (0.11, 0.45)**	−0.20 (− 0.43, 0.04)	−0.16 (− 0.40, 0.08)
Referral						
Intercept	–	1.26 (1.09, 1.43)	1.27 (1.05, 1.50)	–	1.13 (0.97, 1.29)	
Self	**0.14**	**−0.17 (− 0.35, − 0.00)**	**−0.17 (− 0.33, − 0.01)**	−0.12 (− 0.26, 0.02)	−0.05 (− 0.24, 0.14)	

*Sample=participants that had completed at least two sessions and reported having problems longer than six months prior to treatment (*n*= 1059)

Estimates in bold indicate statistical significance at the alpha=.05 level

†Baseline differences were examined in separate analyses and were not part of the latent difference score models

The strongest predictors of *less* improvement on latent symptoms scores were taking antidepressant medication (ES change anxiety − 0.36), being out of work at baseline (ES change depression − 0.18 and ES change anxiety − 0.35), reporting bullying as cause of problems (ES change depression − 0.29), and immigrant background (ES change depression PHQ − 0.27 and ES change anxiety − 0.26), (adjusted estimates). In combination with elevated baseline scores, these observations may indicate that the treatment was less effective for these groups.

Taking sleep medication did on the other hand predict *more* improvement (ES change depression 0.23 and ES change anxiety 0.45, adjusted). Given that we use latent scores and examine a group in which natural recovery is less likely, this might indicate that combining sleep medication and CBT can increase treatment effects.

Some factors predicted *less* improvement (self-referral, physical activity) and some *more* improvement (Job/school-related problems, living alone, relationship problems) with small, adjusted effect sizes only (Table 4). Seen together with deviant latent baseline scores, in respectively the same directions as for the change scores, regression to the mean might be an important alternative explanation for these predictors.

A sensitivity analysis, including treatment duration and number of sessions, respectively, in the multivariate predictor models, slightly reduced the predictive power of some of the predictors and increased others (ES ± 0.01–0.05, details not shown). Accounting for treatment duration and number of sessions did however not change any conclusions.

The remaining baseline characteristics did not predict change in neither latent depression nor anxiety scores. Accordingly, the lower educated showed similar degree of improvement as the higher educated, though notably reporting higher symptom severity both at pre and post treatment (ES baseline score depression 0.42 and anxiety 0.23). The same pattern was found for those reporting difficult childhood experiences as cause of symptoms (ES baseline score depression 0.27 and anxiety 0.43), whereas the opposite was found for level of social support (ES baseline score depression − 0.47 and anxiety − 0.21).

## Discussion

### Main findings

Overall, the results from the 12 first pilot sites of PMHC in Norway indicate that the service was successfully implemented. The clinical results of PMHC were promising as indicated by the large improvements from pre- to post-treatment for symptoms of both depression and anxiety. However, some groups of clients showed less improvement during treatment than others, most notable those having immigrant background, being out of work at baseline, taking antidepressant medicine and reporting bullying as cause of problems. Regarding implementation, key positive features of PMHC were short waiting times and short treatment duration, and that almost half of the participants chose to contact PMHC directly without GP referral. All of this was in line with the guidelines set out by the Norwegian Directorate of Health [14]. Despite the low-threshold features, some groups were under-represented among the clients, namely males, older individuals (> 67 years), those with lower education, and immigrants. Aligning with what is reported from IAPT [10], the majority of participants that contacted PMHC had been struggling with similar psychological problems for a period longer than six months prior to the initial contact, suggesting that there is an urgent need for a service like PMHC.

### Interpretation of clinical outcomes

The effect sizes for PMHC were comparable to those found in the first IAPT pilot sites [10, 11]. Using the same analytic approach (e.g. attending at least two sessions and using LOCF missing data technique) the pre to post effect sizes for PHQ and GAD were respectively 0.9 and 0.8 in PMHC and 1.0–1.2 and 1.1–1.2 in the first two pilots in IAPT. Also in terms of recovery rates, the estimates were solid, comfortably exceeding the 50% recovery target used in IAPT, similar to the overall IAPT recovery rate [11], and far beyond the 5–20% natural recovery found among wait-list control clients with pre-treatment duration of above six months [32–34]. As previously discussed [15], direct comparison with IAPT is difficult. Most important, the lower PHQ and GAD pre-treatment mean scores at PMHC make it easier to fall below the cutoff value for recovery during the course of treatment. Whereas 77% of the PMHC participants were classified as a clinical case at the start of treatment, the number was 92.4% in the latest annual report [12]. In part, this may be because the baseline questionnaire was completed prior to the first treatment sessions and not during the initial assessment. Some clients might therefore already have started an improvement process [43], leading to an underestimation of change scores for PMHC. On the other hand, our definition of “at least two sessions” do not include the initial assessment. This may slightly overestimate the change scores reported in the current study, when comparing to the IAPT results. Finally, the relatively low study participation rate and more missing outcome data in the PMHC sample may introduce nonparticipation bias. Due to all the uncertainties hampering the use of benchmarks from other clinical populations, the initiated randomized controlled trial in PMHC (ClinicalTrials.gov Identifier: NCT03238872) will be of great value to demonstrate more precisely the effect attributable to the treatment provided at PMHC.

### Predictors of change

Regarding the predictors, both demographic (marital status, ethnicity, job status), life style (physical activity) and clinical (medication, perceived cause of symptoms) characteristics contributed in explaining variations in degree of clinical improvement, with small to moderate effect sizes. First, it is worth noting that the improvement was substantial across all groups, as indicated by effect sizes > 0.8 when subtracting the predictor levels from the intercept levels of change. By study design it is difficult to fully disentangle the extent these variations in improvement reflects the differences in response to treatment from regression to the mean effects. The effect of natural recovery was presumably reduced by restricting the analyses to those having long-lasting conditions [32–34], and measurement error was excluded by employing latent variables. Regression to the mean can however not be ruled out as a (partly) alternative explanation of the predictors that showed elevated pre-treatment scores in combination with small deviances in change scores (living alone, intimate partner relation problems, school/job related problems).

Having immigrant background, being out of work, experiencing bullying as cause of problems and daily taking antidepressant medications nonetheless stood out as probable predictors of poorer treatment response. These were all associated with less improvement in symptom scores, most of them with moderate effect sizes while also being associated with higher baseline scores. The first three all represent “structural” factors or problems, which are more or less out of control of the client. A conceivable interpretation is thus that the individual-focused treatment provided through CBT, not sufficiently meet the needs of these clients. Unfortunately, we do not have elaborated information to support this interpretation, for instance, whether the bullying is ongoing or not. Alternative or additional interpretations are that these groups had problems that were more complex and/or were more vulnerable in general [44, 45], and were in need of a more comprehensive treatment than provided within the frames of the PHMC. These interpretations may also apply well, in reverse, to the group reporting job- or school-related problems, as these might be seen as a relative resourceful group with perhaps less complex problems. This group was associated with increased improvement in symptoms of depression. Additionally, during the implementation of PMHC, the health benefits of work was highlighted both by the Directorate of Health and through the therapist curriculum. The therapist might thus have had more competence in and awareness on addressing work-related problems as compared to, for instance, bullying. Interviews with clients and therapist provide mixed support for such interpretation, as the therapists regarded work as an integrated and natural topic during treatment whereas clients noted little focus on work [35].

Regarding immigrants, bearing in mind their heterogeneous backgrounds [45], additional explanations of poorer treatment response include communication difficulties between the therapist and the client, and contradictions between the client’s and the therapist’s conceptions of the illness [46]. In concert with the findings that immigrants both were under-represented and had higher pre-treatment symptom scores as compared to Norwegians, this underscores that the service should take measures to lower barriers for access and better meet the needs of these clients.

Interestingly, self-referral predicted slightly less improvement (ES -0.17) in symptoms of depression. Possible explanations include differences in motivation [47] or group composition, though notably no baseline differences were observed between the self- and health personnel referred other than lower pre-treatment symptom scores among the former. In comparison no differences in outcome were found between the self- and the GP-referred in the first pilots in IAPT [11].

### Implementation efforts

vAltogether, the PMHC sites have accomplished a lot during the pilot period. PMHC was a complex service to implement, especially in the early phase of the pilot project. This was mainly due to the many requirements the pilot sites were expected to meet. These involved the drafting of new procedures; design of intake forms, information material and websites; education and supervision of staff; implementation of job-focused treatment and symptom measurements during treatment; participation in the research project; development of guided self-help materials and procedures; development or adaptation of group courses; and collaboration with other services [35]. It was therefore not beyond expectation that some variation arose with regard to how the pilot sites met requirements, and that some requirements were underprioritized. As a result of good leadership and high levels of professional commitment of the involved therapists, all teams managed to build a new service that was able to provide treatment for people with mild to moderate anxiety and depression.

Despite the abovementioned indicators of PMHC succeeding in being a low-threshold service, men, older individuals, those with low education and immigrant background were underrepresented compared to the population statistics of the PMHC pilot sites. This mirrors what was found at the IAPT pilot sites [11]. The option of self-referral did not increase participation of underrepresented groups. In fact did immigrants use self-referral to a lesser extents than non-immigrants. This contrasts the experience from IAPT [10], but is line with a registry-based study finding that immigrants in Norway are less likely to seek help for mental health problems [48]. Amount of self-referral varied substantially between the pilot sites, presumably partly explained by some being hesitant of broad promotion of their service through the establishing phase. Though such pragmatic solutions might be necessary temporarily, an unintended pitfall may be that it contributes to an inequity of access.

The use of low-intensity treatment was underutilized in many of the PMHC pilot sites, despite previous studies indicating that for example guided self-help can provide similar results as traditional face-to-face treatment [49, 50]. The underutilization may partly be due to the fact that therapists were primarily trained to use face-to-face treatment. In connection with this, guided self-help programs and group courses for psychoeducation were not available at the start of the pilot project. Consequently, many pilot sites had to use considerable amounts of time and resources to develop their own material. This delayed and sometimes even hampered the implementation and subsequent use of low-intensity treatments. It is essential for the further development of PMHC that standardized, evidence-based programs for guided self-help and courses are made available. Both with regard to the desired upscaling of psychological treatment for anxiety and depression, and for the cost-effectiveness of PMHC, increased application of low-intensity treatments is warranted.

### Strengths and limitations

A number of strengths and limitations should be mentioned. Strengths of the study included the relatively large sample size and the use of multiple assessments during the course of treatment, which resulted in large amounts of follow-up data. The multiple assessments helped to reduce potential bias in PHQ/GAD estimates at post-treatment. We used a similar design and partly the same instruments as in IAPT, which has eased the comparisons between the two services. The two outcome measures show high internal consistency and data was analyzed using a variety of statistical models, all of which increase our trust in the results. The pragmatic focus provides a demonstration that treatments developed in controlled settings can be deployed at scale within routine health care systems without major loss of effectiveness. Limitations included the use of a single group design. The mentioned ongoing randomized controlled trial within PMHC will therefore be a major contribution for how the observed changes in the PMHC group compare to the changes in a comparable control group. The participation rate for the study was somewhat low (61% versus 97% in the IAPT pilots), which may have had consequences for the representativeness of our sample. We should therefore be somewhat careful to generalize our findings to the entire PMHC population and, as discussed, directly to compare the sample with the IAPT clients. Some of the measures were rather crude. Though avoiding too extensive questionnaires, the drawback was that it precluded a thorough interpretation of the findings regarding for instance bullying and self-referral. Finally, long-term effects were not examined.

## Conclusion

Large improvement in symptoms of anxiety and depressive were reported by clients having received treatment in this newly implemented low-threshold primary care service across Norway. Bearing in mind the lack of a control group, this indicate that this adaption of IAPT is a viable supplement to the existing health services to increase access of effective treatment for people who suffer from symptoms of mild to moderate anxiety and depression. The services at the 12 sites across Norway were mainly implemented according to the guidelines outlined by the Norwegian Directorate of Health. Yet, PMHC is still in an early phase of development, and there is room for improvement in several areas, most notably increased use of low-intensity treatments such as guided self-help and increased use of PMHC by groups that are currently underrepresented.

## Abbreviations

BMI: Body Mass Index
CBT: Cognitive Behavioural Therapy
CI: Confidence Interval
DSM-IV: Diagnostic and Statistical Manual of mental disorders, 4th edition
ES: Effect Size
GAD: Generalized Anxiety Disorder scale
GP: General Practitioner
IAPT: Increased Access to Psychological Therapy
ITT: Intention To Treat sample
LOCF: Last Observation Carried Forward
MI: Multiple Imputation
PHQ: Patient Health Questionnaire
PMHC: Prompt Mental Health Care
